# Weighted Gene Coexpression Network Analysis Identified MicroRNA Coexpression Modules and Related Pathways in Type 2 Diabetes Mellitus

**DOI:** 10.1155/2019/9567641

**Published:** 2019-12-13

**Authors:** Tianyu Feng, Kexin Li, Pingping Zheng, Yanjun Wang, Yaogai Lv, Li Shen, Yang Chen, Zhiqiang Xue, Bo Li, Lina Jin, Yan Yao

**Affiliations:** ^1^Key Laboratory of Organ Regeneration and Transplantation of Ministry of Education, School of Public Health, Jilin University, Changchun, Jilin, China; ^2^Nursing Department, The Second Hospital of Jilin University, Changchun, Jilin, China

## Abstract

**Objective:**

Type 2 diabetes mellitus (T2DM) is a metabolic disease with high incidence, which has seriously affected human life and health. MicroRNA, a short-chain noncoding RNA, plays an important role in T2DM. Identification of meaningful microRNA modules and the role of microRNAs provide a basis for searching potential biomarkers of T2DM.

**Materials and Methods:**

In this study, three newly diagnosed patients with T2DM and three controls were selected for Whole Peripheral Blood RNA Sequencing to establish a microRNA library. Weighted gene coexpression network analysis (WGCNA) was applied to construct coexpression modules and to detect the trait-related microRNA modules; then, KEGG enrichment analysis was performed to predict the biological function of the interest modules, and candidate hub microRNAs were screened out by the value of module membership (MM) and protein-protein interaction (PPI) network.

**Result:**

Four microRNA modules (blue, brown, magenta, and turquoise) were highly associated with the T2DM; the number of miRNAs in these modules ranged from 41 to 469. The Fc gamma R-mediated phagocytosis pathway, Rap1 signaling pathway, MAPK signaling pathway, and Lysosome pathway were common pathways in three of the four modules. RPS27A, UBC, and RAC1 were the top three proteins in our study; their corresponding RNAs were miR-1271-5p, miR-130a-3p, miR-130b-3p, and miR-574-3p.

**Conclusion:**

In summary, this study identified blood miRNAs in human T2DM using RNA sequencing. The findings may be the foundation for understanding the potential role of miRNAs in T2DM.

## 1. Introduction

Diabetes mellitus, characterized by hyperglycemia, is one of the most prevalent metabolic disorders. It is estimated by the International Diabetes Federation's Diabetes Atlas eighth edition that there are about 1 in 11 adults worldwide now with diabetes mellitus, 90% of whom have type 2 diabetes mellitus (T2DM) [1]. The pathogenesis of T2DM has been extensively studied for many years; it is a complex disorder resulting from the interplay of genetic and environmental factors [2]. In recent years, studies on T2DM tend to explore the biological markers of early diagnosis and intervention from the gene level; the role of microRNAs has attracted wide attention.

MicroRNAs are a class of endogenous short noncoding RNAs, which bind to the 3′terminal noncoding region of target gene at the transcriptional level in the form of complete complementation or incomplete complementation to regulate gene expression and exert biological effects in both health and disease [3–4]. Relevant studies have shown that some microRNAs can regulate the synthesis and secretion of insulin and play an important role in blood glucose balance, such as miR-375 and miR-124a [5–6]. Differential expression analysis was used to screen significant genes for most genetic studies. However, this method only focuses on the effect of the single gene, which cannot find the genetic relationships and build the relationships between genes and disease [7]. Fortunately, this problem can be solved by weighted gene coexpression network analysis (WGCNA).

WGCNA identifies interest gene clusters (which we called gene modules) using information from thousands of genes with the greatest changes, or all of them, and makes significant association analysis with traits [8]. Instead of focusing on differentially expressed genes, it not only makes full use of data information but also converts thousands of gene-trait associations into several gene clusters and trait associations, eliminating the problem of multiple hypothesis test corrections. On the basis of its wide application in cancer research [9–11], WGCNA has a good prospect in T2DM.

The aim of this study was to utilize the WGCNA method to construct coexpression modules for the expression data of miRNAs in human biological specimens and to obtain the correlation between different modules and the trait of T2DM. It provided an effective way to explore the role of microRNAs from different perspectives and provided a basis for searching potential biomarkers of T2DM.

## 2. Materials and Methods

### 2.1. Participants

Three patients who were newly diagnosed with T2DM and three controls without T2DM were recruited at the Second Hospital of Jilin University from April to June 2018. All participants were Han Chinese, and all of them were males aged 40-60 years ([Supplementary-material supplementary-material-1]). Patients were diagnosed according to the China Guideline for T2DM: fasting blood glucose (FBG) > 7.0 mmol/L, oral glucose tolerance test (OGTT) two-hour blood glucose > 11.1 mmol/L. Nondiabetic volunteers were from the health examination center of the second affiliated hospital of Jilin University. The inclusion criteria for nondiabetic volunteers were fasting blood glucose (FBG) < 6.1 mmol/L, oral glucose tolerance test (OGTT) two-hour blood glucose < 7.8 mmol/L. All of them had no family history of diabetes and no history of using antiplatelet or antidiabetic agents. All patients with the history of coronary artery disease (CAD), hypertension, atrial fibrillation, myocardial infarction, tumor, acute infectious disease, immune disease, and hematological disease were excluded from the study.

### 2.2. Ethical Approval and Informed Consent

All participants provided written informed consent; the study was approved by the Institutional Review Board of the Jilin University of Public Health, and it always follows the privacy of the participants.

### 2.3. Blood Sample Collection and RNA Sequencing

The blood samples were collected immediately after admission in the DM group, and the control group blood sample collection was performed the next morning after the participants had fasted for ten hours or overnight by tubes containing EDTA. Total RNA was isolated and purified using RNAiso Plus (total RNA extraction reagent) (TAKARA BIO INC., CA, Japan) according to the manual. RNA purity was checked using the NanoPhotometer® spectrophotometer (IMPLEN, CA, USA). RNA concentration was measured using the Qubit® RNA Assay Kit in a Qubit® 2.0 Fluorometer (Life Technologies, CA, USA). RNA integrity was further assessed using the RNA Nano 6000 Assay Kit of the Agilent Bioanalyzer 2100 system (Agilent Technologies, CA, USA).

### 2.4. Quality Control of Raw Sequencing Data

For microRNAs, raw data (raw reads) of fastq format were firstly processed through custom perl and python scripts. In this step, clean data (clean reads) were obtained by removing reads containing ploy-N, with 5′ adapter contaminants, without 3′ adapter or the insert tag, containing ploy A or T or G or C and low-quality reads from raw data. At the same time, Q20, Q30, and GC content of the raw data were calculated. Taken together, all the downstream analyses were based on the clean data.

### 2.5. Weighted Gene Coexpression Network Analysis

Weighted gene coexpression network analysis was a scale-free network analysis method proposed by Peter Langfelder and Steve Horvath in 2008 [7]. In this method, highly correlated genes are clustered to form modules, module eigengenes (MEs) are used to represent the overall expression level of modules, and modules are correlated with external phenotypes for analysis. This method is widely used in data mining of tumor genes and has obvious advantages over traditional differential gene screening.

#### 2.5.1. Data Pretreatment

The data needs to be processed before the WGCNA analysis. The gene and sample data were transformed into the gene×sample matrix. Fewer than five genes with nonzero expression levels were cleared [12].

#### 2.5.2. Weighted Gene Coexpression Network Analysis and Modules

Bioconductor package in the R programming language was used for WGCNA and goodSamplesGenes function to check whether microRNA data met the requirements of network analysis. Pearson correlation coefficient was calculated for all the genes, and an appropriate soft threshold *β* was automatically selected through the pickSoftThreshold function in the WGCNA package. The function of *β* parameter was to amplify the correlation between genes [13]. Pearson correlation coefficient was exponentially weighted with *β*, and the weighted results were converted into an adjacency matrix [14]. Subsequently, we transformed the adjacency matrix into topological overlap matrix and used topological overlap (TOM) to describe the similarity of gene expression, and 1-Tom to represent the heterogeneity between genes. Finally, dynamic tree was used to divide the modules of hierarchical clustering results, and merging the modules with the number of microRNAs < 30 and cutting height < 0.25 [15].

#### 2.5.3. Key Modules and Hub Genes

Above all, the first principal component module eigengenes (MEs) were calculated to express the expression level of the gene module. Then, the strongly correlated modules were determined by Pearson correlation coefficient between the modules and the phenotype of T2DM. The hub gene was determined by calculating the module membership (MM) and gene significance (GS) of the genes in the key modules. Hub microRNAs were selected according to the top 10% MM value in each module.

### 2.6. Functional Interpretation of Gene Modules

The hub genes' information of interest modules was mapped to the DAVID (Database for Annotation, Visualization, and Integrated Discovery) dataset (https://david.ncifcrf.gov/summary.jsp). Then, the functional enrichment analysis was performed by KOBAS3.0 (http://kobas.cbi.pku.edu.cn/index.php) to interpret the biological function of each interest module.

### 2.7. Selection of Hub MicroRNAs and Construction of Protein-Protein Interaction

Module membership (MM) was defined as the correlation of expression profile and MEs. The hub microRNAs were determined by calculating the module membership (MM), and the first 10% according to the value of MM in each module was selected. Subsequently, we used the target genes of hub microRNAs to construct protein-protein interaction (PPI) network by STRING database (https://string-db.org/) to find potential functional proteins and important microRNAs in this study.

### 2.8. Cross Validation

GSE21321 is an independent cohort which include both microRNA and mRNA database [16]. In order to validate the hub RNAs in our study, we only downloaded the microRNA dataset, and excluding seven impaired fasting glucose cases, leaving nine T2DM cases and ten normal controls for follow-up analysis. All 19 participants were Singaporean and males. They were without past history of T2DM and with desirable cholesterol and blood pressure profiles, only classified according to fasting glucose levels to have T2DM (fasting glucose ≥ 7.0 mmol/L) and healthy controls (fasting glucose < 6.1 mmol/L). Data for GSE21321 were obtained by using GPL10322 (v.11.0-hsa, mmu, and rno (probe-level)) miRCURY LNA microRNA array. The background correction, normalization, and summarization were performed using the robust multichip average algorithm. After that, we used the log2 transformation to process the intensities of microRNAs that extracted from the GSE21321. Differential expression analysis was performed by using the *t*-test; the individual *P* values and |Log2FoldChange| were obtained to validate the significance of RNAs we have found.

## 3. Result

### 3.1. Overview of the Transcriptome Profiling

Three T2DM patients and three healthy controls were involved in this study. Blood samples from 6 individuals were extracted for RNA sequencing and analysis; then, a small RNA library was established. In order to ensure the quality of information analysis, low-quality reads with joints were removed; clean reads obtained by sequencing were processed. The clean reads in the T2DM group were 12507829, 12817714, and 11412993, respectively, while those in the control group were 12097060, 11718875, and 13818644, respectively (Table 1). Subsequently, the reads were standardized and transformed into TPM values for subsequent analysis.

### 3.2. Determination of Soft Threshold

**Figure 1:**
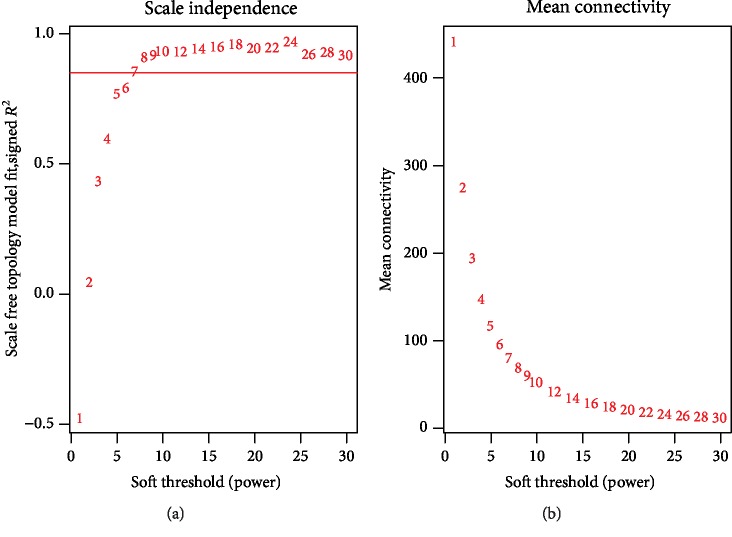
The network topology under different soft thresholds. (a) The chart showed the correlation coefficients of log(k) and log(p(k)) corresponding to different soft thresholds; (b) the chart showed the mean values of gene adjacency coefficients corresponding to different soft thresholds, reflecting the average connectivity level of the network.

According to the WGCNA software package in R, the correlation analysis was carried out. There were 1344 primitive microRNAs, and 958 were left after excluding the nonconforming data. The results show that the coexpression network conform to scale-free network, that is, the logarithmic log (k) of the node with K connectivity is negatively correlated with the logarithmic log(p(k)) of the probability of the node's occurrence, and the absolute value of the correlation coefficient is greater than 0.8. In this study, the correlation coefficient was 0.85, and the soft threshold was 7 (Figure 1). The adjacency matrix and the topological overlap measure (TOM) were further constructed by weighing coefficient. The hierarchical clustering analysis of microRNAs was carried out according to the difference of nodes (1-TOM).

### 3.3. Identifying Coexpression Module and Module Preservation Analysis

**Figure 2:**
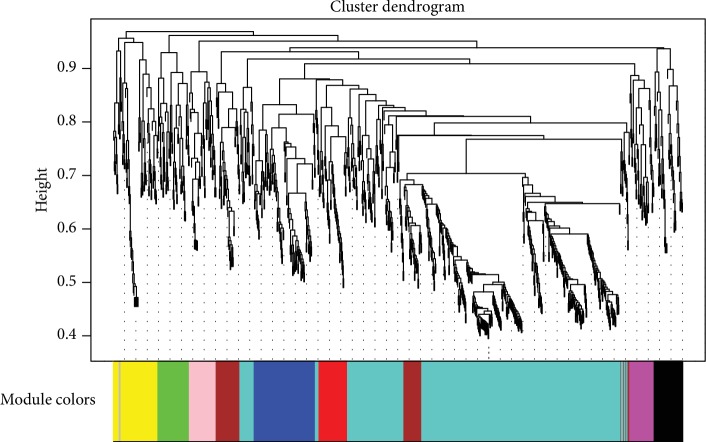
Hierarchical clustering tree and coexpression module of microRNAs. At the top of the graph was a clustering tree of microRNAs, and at the bottom are different modules cut from the dynamic cutting tree (different colors represent different modules).

Figure 2 is a hierarchical clustering tree constructed according to the weight of microRNAs. Modules were classified by dynamic tree cut, and merging the modules with the number of microRNAs was less than 30 and the cutting heights < 0.25. Finally, grey, blue, green, magenta, red, yellow, black, brown, pink, and turquoise (different colors represent different modules) 10 modules were obtained; the number of microRNAs in different modules is displayed in Table 2. The RNAs were contained in the grey module implying that it was not assigned to any module.

**Figure 3:**
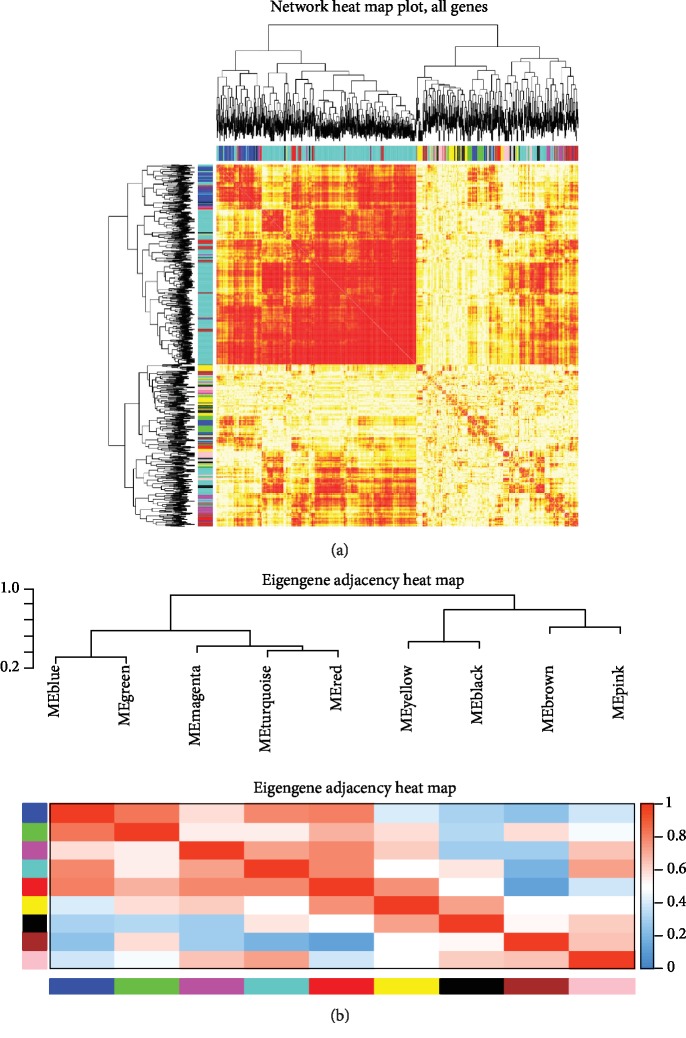
Module preservation analysis. (a) Represented the gene network by using a heat map plot. The heat map depicted the topological overlap matrix (TOM) among all genes in the analysis. Light color represents low overlap, and progressively darker red color represents higher overlap. (b) Represented the heat map plot of the adjacencies of modules. Red represented high adjacency (positive correlation), while blue color represented low adjacency (negative correlation).

The most representative gene set in each module represented the overall level of gene expression in the module as the first principal component of the module eigengene (ME).  Figure 3 represents the correlation between microRNAs. The correlation between modules not only showed the specificity but also showed the biological significance of the miRNA module in this study.

### 3.4. Module-Clinical Trait Relationships

**Figure 4:**
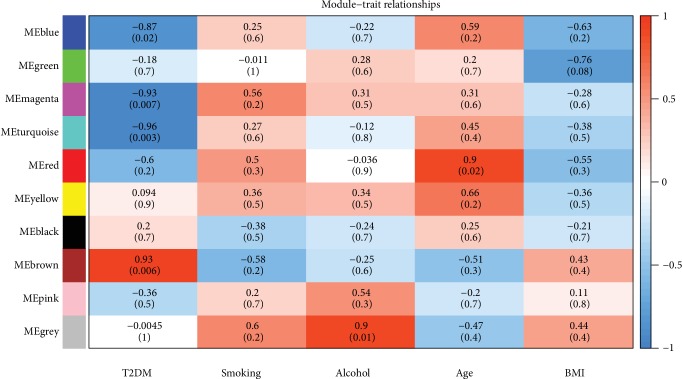
Module-trait associations. Each row corresponded to a module eigengene, column to a trait. Each cell contained the corresponding correlation and *P* value. The cells were color coded by correlation according to the color legend.

Identifying genes associated with a certain clinical trait is of great value to explore the molecular mechanisms behind that trait. In the present study, the parameters of six samples included BMI, age, smoking, alcohol, and T2DM. Since this study explored the mechanism of T2DM, we mainly focused on modules highly related to it. Pearson correlation coefficients between ME and the clinical trait of each microRNA module were calculated, and significance module (*P* < 0.05) was obtained to further screen coexpression modules. As shown in Figure 4, we found four modules were significantly associated with T2DM (blue module, brown module, turquoise module, and magenta module). MEblue correlation coefficient was -0.87; *P* value was 0.02. MEgreen module correlation coefficient was -0.18; *P* value was 0.7. MEmagenta module correlation coefficient was -0.93; *P* value was 0.0017. MEturquoise module correlation coefficient was -0.96; *P* value was 0.003. Except for these four modules, there was no statistical significance in other modules.

### 3.5. Pathway Enrichment Analysis of MicroRNAs

**Figure 5:**
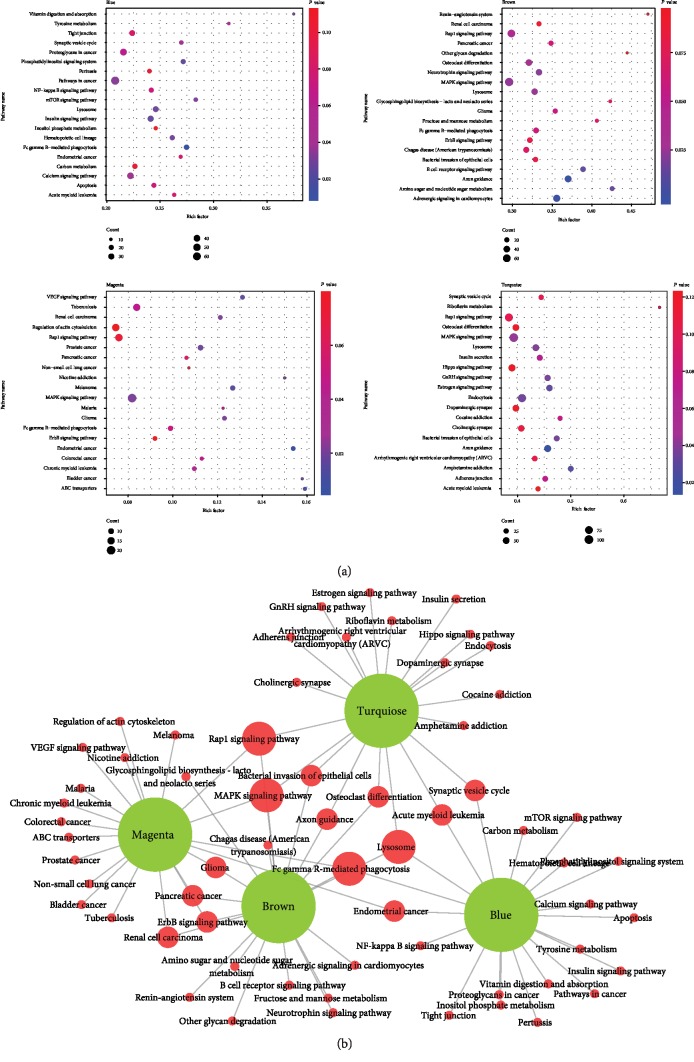
Kyoto Encyclopedia of Genes and Genomes (KEGG) pathway analyses of four significant modules. (a) Four figures represented the first 20 pathways in four modules, respectively. The abscissa was the rich factor; the ordinate was the pathway term. (b) Represented the overlapping parts of the first 20 pathways of the four modules.

To explore the biological functions of four modules, we performed pathway analysis to the target genes corresponding to each module's microRNAs. In Figure 5(a), four bubble charts represented the top 20 pathways of each module and comprehensive analysis of rich factor; count and *P* value found significant pathways. Among four modules, the Rap1 signaling pathway and MAPK signaling pathway were both enriched in magenta, brown, and turquoise modules; the lysosome pathway was enriched in blue, brown, and turquoise modules, while blue, brown, and magenta common pathways were Fc gamma R-mediated phagocytosis (Figure 5(b)). These four pathways might have potential roles in T2DM.

### 3.6. Identification of Hub MicroRNAs and Construction of Protein-Protein Interaction (PPI) Network

**Figure 6:**
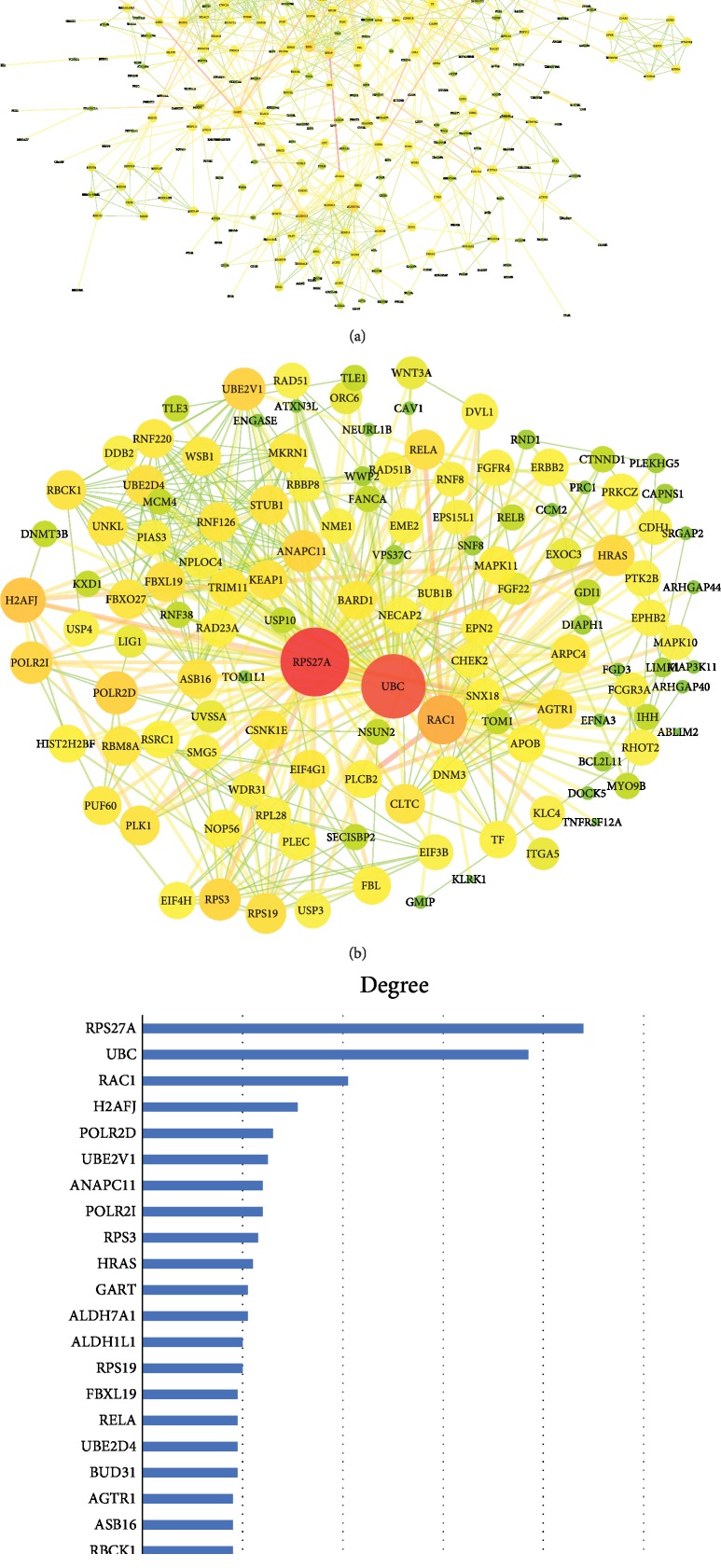
(a) The protein-protein interaction (PPI) analysis of target gene of hub microRNAs. Edge stood for the interaction between two genes. A degree was used for describing the importance of protein nodes (red represented high degree and blue represented low degree); (b) represented the highest degree of PPI in (a); (c) represented the degree of the first 25 proteins.

Module membership (MM) represented each microRNA's Pearson correlation coefficient with corresponding module eigengene. Since RNA of the same module had the same biological function, hub microRNAs according to the top 10% MM value in each module were utilized to represent the biological function of the whole module ([Supplementary-material supplementary-material-1]). Then, the target genes of hub microRNAs were used for protein-protein interaction (PPI) analysis. It could throw light on the study of molecular mechanism of disease from a systematic perspective, finding new drug targets. In PPI network, the top three proteins were RPS27A, UBC, and RAC1, and their degrees were 88, 77, and 41, respectively (Figure 6). For RAC1, we combined the KEGG pathway analysis and found that RAC1 could be involved in 15 signaling pathways. It was worth noting that these 15 pathways were distributed in all four important modules ([Supplementary-material supplementary-material-1]). The corresponding microRNAs of RPS27A were miR-1271-5p, UBC was miR-130b-3p and miR-130a-3p, and RAC1 was miR-574-3p. The four RNAs were key microRNAs which were screened out.

### 3.7. Validation in the GEO Dataset

The expression pattern of 69 hub RNAs was verified using the GSE21321 dataset. RNA sequencing is superior to microarray for characterizing transcriptomes. The hub RNAs of T2DM in this study were obtained by RNA sequencing performed on an Illumina platform. However, GSE21321 data were obtained from GPL10322 miRCURY LNA microRNA array, and the chips used in GSE21321 are of an early age; the microRNA in this array was not enough to detect all microRNAs in this study. 40 hub microRNAs were detected in GSE21321; nine hub RNAs met the differential expression criteria of *P* < 0.1 and ∣Log2FoldChange∣ > 1 (Table 3). Four key RNAs that were screened by PPI network could also be validated by GSE21321 dataset. Among them, the *P* value of miR-574-3p was 0.011 and Log2FoldChange was 1.205. Although the expressions of miR-1271-5p, miR-130a-3p, and miR-130b-3p in GSE21321 had no statistical significance, it cannot be denied that these three microRNAs may play an important role in the development of T2DM.

## 4. Discussion

In this study, ten modules were obtained by WGCNA analysis, out of which four of them were highly related to T2DM. Blue, magenta, and turquoise modules were negatively correlated with the T2DM, while brown module was positively correlated with the T2DM.

In the top 20 KEGG pathways of the four modules, it was worth noting that Fc gamma R-mediated phagocytosis, lysosome, MAPK signaling pathway, and Rap1 signaling pathway were common pathways.

MAPK signaling pathway contains 257 genes. The mitogen-activated protein kinase (MAPK) pathway is sensitive to a variety of inflammatory cytokines. In insulin resistance, there is usually a chronic inflammatory response, which makes the MAPK pathway play an important role in accompanying the inflammatory response of type 2 diabetes, such as diabetic nephropathy and liver lesions. However, the functional role of MAPKs in insulin resistance has not been clarified; the specific mechanism of the MAPK pathway in type 2 diabetes needs to be further studied [17–21].

The Rap1 signaling pathway contains 211 genes. Rap1 is a small GTPase that controls a variety of processes, including cell adhesion and connection formation. Previous studies have shown that the circulating microRNAs of type 1 diabetes mellitus are enriched by the Rap1 signaling pathway [22]. By inhibiting VEGF signal transduction, Rap1B can prevent excessive vascular leakage in early diabetes mellitus. By controlling telomere length, Rap1 can prevent the occurrence and development of diabetes-related cardiovascular disease [23]. Inhibition of Rap1B to reduce VEGF signal transduction can prevent excessive vascular leakage in early diabetes mellitus [24]. At the same time, Rap1 also regulates MAP kinase (MAPK) activity in a manner highly dependent on the context of cell types [25]. Although numerous studies have been conducted on the mechanism of the Rap1 signaling pathway in diabetes, there are no studies on related microRNAs.

The lysosome pathway contains 122 genes. Lysosomes are membrane-delimited organelles in animal cells serving as the cell's main digestive compartment. Their functions include endocytosis, phagocytosis, and autophagy. Many studies had pointed out that lysosomes play a key role in T2DM and its complications through autophagy and phagocytosis. Autophagy dysfunction was an important factor in triggering T2DM. Autophagy can maintain the normal cell structure of islet B cells, while excessive autophagy can lead to a decrease in number of islet B cells [26–28]. Therefore, the regulation of autophagy function might become a new target for the treatment and prevention of diabetes, and the related microRNAs might become the key to the regulation of autophagy function.

Fc gamma R-mediated phagocytosis contains 91 genes Fc *γ* receptors (Fc*γ*Rs) are receptors for IgG that classically regulate processes in immune response [29]. Experiments in mice showed that c-reactive protein activated the endothelial cells Fc RIIB which might have a negative effect on the glucose balance. Blocking Fc R can reduce the occurrence of type 2 diabetes [29, 30]. Therefore, Fc R may be the key to the prevention and treatment of type 2 diabetes. Due to the lack of relevant studies, the mechanism of Fc R's participation in type 2 diabetes remains to be further studied [31].

In addition to KEGG enrichment analysis of key modules, STRING database was used to conduct protein-protein interaction (PPI) network of some hub genes in key modules. By PPI network analysis, RPS27A, UBC, and RAC1 have the highest degree value in network. An exploratory study had demonstrated that RPS27A may be a potential target for mesenchymal stem cells in the treatment of T2DM [32]. Some studies had shown that miR-130a-3p and miR-130b-3p could affect the occurrence and development of T2DM by reducing the ATP level in pancreatic islet cells [33], and UBC was regulated by these two microRNAs. Despite the lack of studies on the role of RPS27A and UBC in T2DM, ubiquitin proteins play an important role in the occurrence and development of T2DM [34]. In future studies, the pathways and mechanisms of these two proteins and the microRNA affecting their expression can be further studied.

In order to explore the role of RAC1, we tried to find out the correlation between RAC1 and the KEGG enrichment pathway of the module. RAC1 was involved in 15 of these pathways. Among them, the MAPK signaling pathway and Rap1 signaling pathway were also important common pathways in KEGG enrichment analysis of the modules. Notably, many previous studies had attempted to elucidate the association between RAC1 and diabetes and the molecular mechanisms involved [35–37]. RAC1 was a small guanosine triphosphate- (GTP-) bound protein belonging to the Rho family. Numerous studies had demonstrated the important role of RAC1 in various stages of T2DM [38, 39]. In this study, miR-574-3p was the hub gene in the turquoise module [40]. Therefore, we believed that miR-574-3p and RAC1 might be potential biomarkers or new therapeutic targets for diabetes.

## 5. Limitation

Due to the small sample size used in this study, the results of this study might be limited when extrapolated. At the same time, this study was an exploratory study, comprising of only a small number of T2DM patients and nondiabetic participants; the results need to be verified by experiments for the next step.

## 6. Conclusion

In this study, four microRNA modules related to T2DM were identified through WGCNA. In-depth analysis of the four modules, four microRNAs: miR-1271-5p, miR-130a-3p, miR-130b-3p, and miR-574-3p and three proteins: RPS27A, UBC, and RAC1, was identified by KEGG enrichment analysis and PPI network. RAC1 and miR-574-3p were most likely to be biomarkers of T2DM by combining KEGG enrichment analysis and PPI network. The findings may be the foundation for understanding the potential role of miRNAs in T2DM.

## Figures and Tables

**Table 1:** Summary of data cleaning from RNA sequencing.

Sample	Total reads	N% > 10%	Low quality	5 adapter contamine	3 adapter null or insert null	With ployA/T/G/C	Clean reads
B1	12778191 (100.00%)	(0.00%)	122139 (0.96%)	266 (0.00%)	358460 (2.94%)	2474 (0.02%)	11832483 (96.94%)
B2	13107612 (100.00%)	(0.00%)	137294 (1.05%)	308 (0.00%)	318292 (2.70%)	2307 (0.02%)	11434838 (97.18%)
B3	11677326 (100.00%)	(0.00%)	126144 (1.08%)	172 (0.00%)	326354 (2.62%)	3502 (0.03%)	12107635 (97.21%)
D1	12476427 (100.00%)	(0.00%)	23555 (0.19%)	128 (0.00%)	295178 (2.37%)	2492 (0.02%)	12155074 (97.42%)
D2	12165981 (100.00%)	6 (0.00%)	19821 (0.16%)	270 (0.00%)	354952 (2.92%)	2703 (0.02%)	11788229 (96.90%)
D3	14285486 (100.00%)	113 (0.00%)	17783 (0.12%)	208 (0.00%)	359335 (2.52%)	3962 (0.03%)	13904085 (97.33%)

**Table 2:** Number of microRNAs contained in different modules.

Module	Blue	Green	Magenta	Red	Yellow
Number	103	53	41	48	72
Module	Black	Brown	Pink	Turquoise	Grey
Number	48	76	45	469	3

**Table 3:** Hub microRNAs validated in the GSE21321.

ID	MicroRNA	*P* value	Log2FoldChange
4610	miR-126-5p	0.075	5.196
42640	miR-20b-5p	0.094	1.375
31026	miR-101-3p	0.056	3.986
30687	miR-93-5p	0.092	4.723
17280	miR-15b-3p	0.054	9.758
10998	miR-19b-3p	0.099	2.422
17565	miR-30b-5p	0.091	6.295
28966	miR-574-3p	0.011	1.205
11039	miR-29a-3p	0.092	5.211

## Data Availability

The datasets generated or analyzed during the current study are available from the corresponding author (Yan Yao, email: yaoyan@jlu.edu.cn) on a reasonable request.

## Supplementary Materials

Supplementary Materials Table 1: descriptive characteristics of participants. Table 2: hub microRNAs in four modules. Table 3: pathway of RAC1 participation.
