# Supplementary material for: Weighted Gene Coexpression Network Analysis Identified MicroRNA Coexpression Modules and Related Pathways in Type 2 Diabetes Mellitus
Source: Oxid Med Cell Longev. 2019 Dec 13;2019:9567641. doi: 10.1155/2019/9567641 (PMC6935443; doi:10.1155/2019/9567641)
Supplement: Supplementary Materials — Table 1: descriptive characteristics of participants. Table 2: hub microRNAs in four modules. Table 3: pathway of RAC1 participation. [file 9567641.f1.docx]

**Table 1** descriptive characteristics of participants

|  | Case1 | Case2 | Case3 | Control1 | Control2 | Control3 |
| --- | --- | --- | --- | --- | --- | --- |
| Race | Asian | Asian | Asian | Asian | Asian | Asian |
| Age | 40 | 45 | 55 | 51 | 51 | 52 |
| DM | Yes | Yes | Yes | No | No | No |
| Hypertension | No | No | No | No | No | No |
| Smoking status | No | No | No | No | No | Yes |
| Alcohol consumption | No | Yes | No | No | No | Yes |
| Height(cm) | 172 | 175 | 173 | 170 | 172 | 169 |
| Weight(kg) | 98 | 80 | 70 | 60 | 76 | 80 |

**Table 2** Hub microRNAs in four modules

| Turquoise | Blue | Brown | Magenta |
| --- | --- | --- | --- |
| miR-6511b-3p | miR-627-5p | miR-10a-5p | miR-28-3p |
| miR-378a-3p | miR-5189-3p | miR-636 | miR-939-5p |
| miR-191-5p | miR-3667-5p | miR-4664-3p | miR-3115 |
| miR-1271-5p | miR-32-5p | miR-4473 | miR-500b-3p |
| miR-130b-3p | miR-29a-3p | miR-659-5p |  |
| miR-181a-3p | miR-221-3p | miR-3158-3p |  |
| miR-126-5p | novel_612 | miR-3158-5p |  |
| miR-210-3p | miR-4685-3p | miR-4672 |  |
| miR-20b-5p | miR-4678 |  |  |
| miR-101-3p | miR-4659b-3p |  |  |
| miR-29b-2-5p |  |  |  |
| miR-548t-3p |  |  |  |
| miR-491-5p |  |  |  |
| miR-93-5p |  |  |  |
| miR-15b-3p |  |  |  |
| miR-548k |  |  |  |
| miR-1287-5p |  |  |  |
| miR-1306-5p |  |  |  |
| miR-5188 |  |  |  |
| miR-19b-3p |  |  |  |
| miR-324-3p |  |  |  |
| miR-144-3p |  |  |  |
| miR-130b-5p |  |  |  |
| miR-16-5p |  |  |  |
| miR-942-5p |  |  |  |
| miR-30b-5p |  |  |  |
| miR-548ap-3p |  |  |  |
| miR-92a-3p |  |  |  |
| miR-4732-3p |  |  |  |
| miR-378c |  |  |  |
| miR-532-3p |  |  |  |
| miR-548ad-5p |  |  |  |
| miR-20a-5p |  |  |  |
| miR-194-5p |  |  |  |
| miR-197-3p |  |  |  |
| miR-29c-5p |  |  |  |
| miR-24-2-5p |  |  |  |
| miR-92b-3p |  |  |  |
| miR-574-3p |  |  |  |
| miR-2110 |  |  |  |
| miR-550a-3p |  |  |  |
| miR-550b-2-5p |  |  |  |
| miR-4732-5p |  |  |  |
| miR-7976 |  |  |  |
| miR-362-5p |  |  |  |
| miR-6511a-3p |  |  |  |
| miR-362-3p |  |  |  |

**Table 3** Pathway of RAC1 Participation

| RAC1 | Blue | Fc gamma R-mediated phagocytosis |
| --- | --- | --- |
|  |  | Pathways in cancer |
|  |  | Proteoglycans in cancer |
|  |  | Tight junction |
|  | Brown | Axon guidance |
|  |  | B cell receptor signaling pathway |
|  |  | Bacterial invasion of epithelial cells |
|  |  | Fc gamma R-mediated phagocytosis |
|  |  | MAPK signaling pathway |
|  |  | Neurotrophin signaling pathway |
|  |  | Osteoclast differentiation |
|  |  | Rap1 signaling pathway |
|  |  | Renal cell carcinoma |
|  | Magenta | Colorectal cancer |
|  |  | Fc gamma R-mediated phagocytosis |
|  |  | MAPK signaling pathway |
|  |  | Rap1 signaling pathway |
|  |  | Regulation of actin cytoskeleton |
|  |  | Renal cell carcinoma |
|  |  | VEGF signaling pathway |
|  | Turquoise | Axon guidance |
|  |  | Bacterial invasion of epithelial cells |
|  |  | MAPK signaling pathway |
|  |  | Osteoclast differentiation |
|  |  | Rap1 signaling pathway |
